# The dynamic action of SecA during the initiation of protein translocation

**DOI:** 10.1042/BJ20121314

**Published:** 2013-01-09

**Authors:** Vicki A. M. Gold, Sarah Whitehouse, Alice Robson, Ian Collinson

**Affiliations:** School of Biochemistry, University of Bristol, University Walk, Bristol BS8 1TD, U.K.

**Keywords:** ATPase, membrane–protein dynamics, protein–translocation, SecA, Sec complex, C_12_E_9_, nona(ethylene glycol) dodecyl ether, CL, cardiolipin, DDM, dodecyl maltoside, DTT, dithiothreitol, 2HF, two-helix finger, HSD, helical scaffold domain, HWD, helical wing domain, NBD, nucleotide-binding domain, p[NH]ppA, adenosine 5′-[β,γ-imido]triphosphate, PPXD, pre-protein cross-linking domain, TMS, transmembrane segment

## Abstract

The motor ATPase SecA drives protein secretion through the bacterial Sec complex. The PPXD (pre-protein cross-linking domain) of the enzyme has been observed in different positions, effectively opening and closing a clamp for the polypeptide substrate. We set out to explore the implicated dynamic role of the PPXD in protein translocation by examining the effects of its immobilization, either in the position occupied in SecA alone with the clamp held open or when in complex with SecYEG with the clamp closed. We show that the conformational change from the former to the latter is necessary for high-affinity association with SecYEG and a corresponding activation of ATPase activity, presumably due to the PPXD contacting the NBDs (nucleotide-binding domains). In either state, the immobilization prevents pre-protein transport. However, when the PPXD was attached to an alternative position in the associated SecYEG complex, with the clamp closed, the transport capability was preserved. Therefore large-scale conformational changes of this domain are required for the initiation process, but not for translocation itself. The results allow us to refine a model for protein translocation, in which the mobility of the PPXD facilitates the transfer of pre-protein from SecA to SecYEG.

## INTRODUCTION

Protein secretion and membrane insertion occur through the ubiquitous SecY/61 complex, driven by associated ribosomes or motor proteins. Secretory proteins are recognized by virtue of cleavable signal sequences at the N-terminus and by elements contained in the mature protein [1,2]. In bacteria, the ATPase SecA targets pre-secretory proteins to SecYEG at the cytosolic membrane and drives translocation through it [3]. The structures of these components are known [4–7], but major questions lie in the nature of the dynamic interaction between the translocation machinery and its substrate.

The protein channel is formed between two pseudo-symmetrical halves of SecY, comprising TMSs (transmembrane segments) 1–5 and 6–10 [5]. This arrangement also allows the movement of membrane protein segments laterally into the lipid bilayer, by passing between TMSs 2b and 7 of SecY; this lateral gate also forms the signal-sequence-binding site [5,8]. Protein translocation proceeds through one of the SecYEG complexes contained in the functional dimeric translocon, whereas the other copy helps to provide a high-affinity binding platform for SecA [9,10]. Recent work *in vivo* suggests that this dimeric interaction may change or be lost during translocation [11].

**Figure 1:**
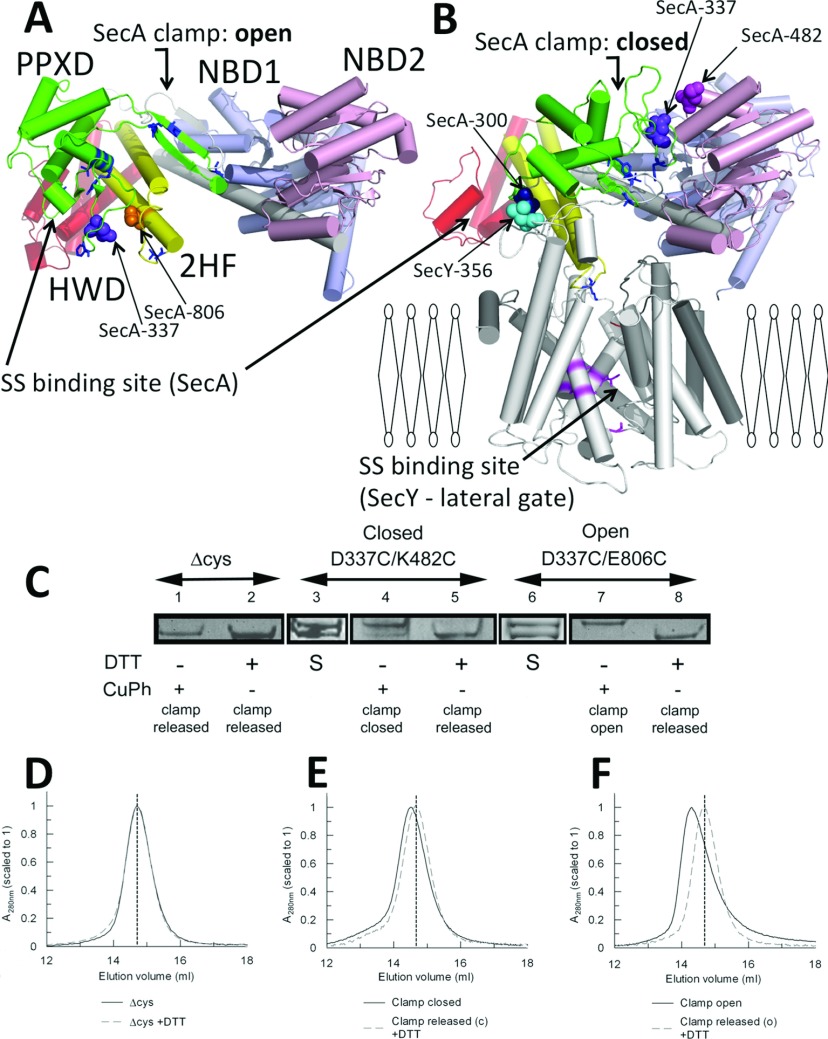
Interactions of the PPXD SecA protomer from a structure determined as (**A**) a dimer [6] and (**B**) a monomer bound to SecYEG (white) [7], viewed from the side of the membrane, with the α-helices shown as cylinders. NBD1 is shown in blue, NBD2 in pink, the HSD including 2HF in yellow, the PPXD in green and the HWD in red. The signal-sequence-binding sites are shown for SecA [15] and SecY [20]. Specific residues used for cross-linking are shown in space-fill representation, and labelled according to the *E*. *coli* residue numbers: SecA_D337C/E806C_ (clamp open; depicted in **A**), SecA_D337C/K482C_ (clamp closed; depicted in **B**), SecA_300_–SecY_356_EG (clamp closed; depicted in **B**). (**C**) Gradient SDS/PAGE was used to resolve the intramolecular cross-links in the presence of oxidizing (1 mM copper phenanthroline) or reducing (10 mM DTT) agent. Lanes 1 and 2, SecA_Δcys_; lanes 3–5, SecA_D337C/K482C_; lanes 6–8, SecA_D337C/E806C_. S denotes the starting material, which contained both species [cross-linked (top band) and uncross-linked (bottom band)]. (**D**–**F**) The corresponding size-exclusion chromatography analysis for each sample is shown below: (**D**) SecA_Δcys_±DTT; (**E** and **F**) cross-linked and uncross-linked mutants (black continuous traces or grey broken traces respectively) used to trap the clamp in the closed and open states. A vertical broken line shows the elution volume of SecA_Δcys_ in all three traces. CuPh, copper phenanthroline.

In solution SecA is dimeric [12]. Each monomer can be divided into five domains: two NBDs (nucleotide-binding domains; NBD1 and NBD2), between which ATP is bound and hydrolysed, an HSD (helical scaffold domain), coupling the NBDs to the HWD (helical wing domain) and the PPXD (pre-protein cross-linking domain) [6,13–15] (Figures 1A and 1B). The latter is thought to play a dynamic role during the activation of the enzyme [16]. Monomer formation results in PPXD relocation towards the NBDs [17], resulting in the perturbation of the signal-sequence-binding site formed between the PPXD and the HWD [14].

The association of SecA with SecYEG has major consequences on the structure and activity. Dimers dissociate and the PPXD swings all the way from the HWD to contact NBD2 [7,18] (Figures 1A and 1B). Another result is the penetration of a 2HF (two-helix finger) of SecA into the protein channel [7]. These events correspond to a major shift in the kinetic properties of the enzyme: a 160-fold reduction in the affinity (increase in *K*_m_) for ATP and a 30-fold stimulation in the ATPase activity by alleviation of the rate-limiting step, the release of ADP [19]. The distance between distinct signal-sequence-binding sites in SecA and SecYEG is approximately 60 Å (1 Å=0.1 nm) [7] (Figure 1B) and the mechanism of its transfer from one to the other is unknown.

The route for translocating pre-protein has been mapped by cross-linking the substrate proOmpA to SecY [20] and SecA [15]. The path runs through two coincident channels formed by a ‘clamp’ between the PPXD and NBD2 of SecA and the channel through the centre of SecY. The PPXD has been proposed to assist in translocation by successively trapping and releasing polypeptide in the clamp during ATP hydrolysis, together with the concerted insertion and retraction of the 2HF [7,21].

In the present study we devised a series of experiments to evaluate the dynamic action of the PPXD throughout the ATP hydrolysis cycle and protein translocation. On the basis of the structure of SecA and its complex with SecYEG, cysteine pairs were designed to immobilize the PPXD in the position occupied in the SecA dimer with the clamp in the open position [6], and in the SecYEG-bound state with it closed [7] (Figures 1A and 1B). Engineered proteins incorporating intramolecular SecA disulfide cross-links, or intermolecular SecA–SecYEG cross-links were used to correlate the consequences of the position and mobility (or immobility) of the PPXD to ATP hydrolysis and pre-protein transport. The results of the present study demonstrate that the relocation of the PPXD towards NBD2 is necessary for the initial activation of the ATPase high-affinity association with SecYEG and intercalation of pre-protein. The present study complements our recent analysis of the role of the 2HF in protein translocation [22].

## MATERIALS AND METHODS

### Chemicals and biochemicals

All lipids were purchased from Avanti. Detergents were from Glycon. BioBeads and gel-filtration standards were from Bio-Rad. NuSep pre-cast gels were supplied by Generon, and all chromatographic material was from GE Healthcare Life Sciences. The QuikChange® kit was from Agilent Technologies and all other reagents were acquired from Sigma.

### Cloning, expression and purification of the wild-type translocation components

Cloning, expression and purification of the translocation components and specific mutants thereof were conducted as described previously [9,23]. Reconstitution of SecYEG into total *Escherichia coli* polar lipids has been well documented [9]. The proOmpA used was a cysteine-less construct [19] designated proOmpA_Δcys_.

### Cross-linking and purification of SecA with the clamp in open or closed positions

Double-cysteine mutants SecA_D337C/E806C_ (clamp open) and SecA_D337C/K482C_ (clamp closed) were introduced into cysteine-less SecA in the plasmid pT7N95-SecA(C98S) [17] using the QuikChange® site-directed mutagenesis kit. After expression in *E. coli* using the standard protocol [23], the intramolecular cross-links were present at approximately 50% of the total material. Following the nucleotide-stripping procedure [23], cross-links were further oxidized by incubation with 1 mM copper phenanthroline for 1 h at room temperature (21°C). This procedure was also followed for the SecA_Δcys_ control as a mock treatment. To purify the cross-linked material away from the uncross-linked material, SecA was exchanged into 20 mM Mes (pH 6) and 100 mM KCl and subjected to cation exchange (Mono S HR 16/10). Proteins were eluted with a 200 ml linear gradient of 0.1–1 M KCl at 2 ml/min and resolved on 4–20% NuSep pre-cast gels. The copper-phenanthroline-treated cross-linked material (and the SecA_Δcys_ mock preparation) were then subjected to size-exclusion chromatography (Superdex 200 HR 26/60) in gel-filtration buffer [20 mM Tris/HCl (pH 7.5) and 100 mM KCl]. The uncross-linked material (and the remaining half of the SecA_Δcys_ mock preparation) was treated in the same way, but with the inclusion of 10 mM DTT (dithiothreitol) in the final chromatographic buffer.

### Analytical size-exclusion chromatography of SecA with the clamp in open or closed positions

Size-exclusion experiments were conducted on a Superose 6 10/300 GL column in SecA gel-filtration buffer, with or without 10 mM DTT. The column was calibrated in both buffers using gel-filtration standards to gain values for apparent molecular masses.

### Purification of cross-linked SecA_300_–Y_356_EG

Total membranes extracted from 12 litres of *E. coli* overexpressing SecY_I356C_EG were resuspended in 100 ml of TSGM buffer [20 mM Tris/HCl (pH 8), 130 mM NaCl, 10% glycerol and 2 mM MgCl_2_]. In each case 100 nmol of SecA_S300C_ was added together with 25 μM p[NH]ppA (adenosine 5′-[β,γ-imido]triphosphate), before oxidation with 0.6 mM copper phenanthroline for 45 min at 4°C. The oxidizing agent was then removed by dialysis before the membranes were re-isolated by centrifugation. The oxidized membranes were then washed twice in 100 ml of TSG to help remove the uncross-linked excess SecA. The membranes were then solubilized in TSGM buffer including 2% (w/v) DDM (dodecyl maltoside) for 1 h at 4°C, and the insoluble material was removed by centrifugation. The cross-linked complexes were then purified by successive Ni^2+^-chelating Q-Sepharose high-performance and size-exclusion chromatography in a manner similar to that used previously for the wild-type and mutant SecYEG complexes [19]. As before, the uncross-linked SecYEG complexes washed through the Q-column (GE Lifesciences; XK 16/10, ~20 ml bed volume) in TSGM buffer with 130 mM NaCl and 0.1% C_12_E_9_ [nona(ethylene glycol) dodecyl ether]. The bound cross-linked SecA–SecYEG and free SecA were eluted separately by a NaCl gradient (130–1000 mM in 100 ml) in TSGM buffer with 0.1% DDM. The appropriate fractions were pooled and further purified by gel filtration using a Superdex 200HR XK16/60 in TSGM buffer (130 mM NaCl) with 0.1% DDM (see Figures 6A and 6B).

### Steady-state ATPase and protein translocation assays

Steady-state SecA ATPase measurements were performed as described previously [9,19]. Briefly, activity was assayed using a Lambda 25 spectrophotometer (PerkinElmer) in TKM buffer [50 mM triethanolamine, 50 mM KCl and 2 mM MgCl_2_ (pH 7.5)] containing 1 unit of pyruvate kinase, 1.4 units of lactate dehydrogenase, 0.2 mM NADH and 2 mM phosphoenol pyruvate, with or without 10 mM DTT in 100 μl cuvettes at 25°C. Detergent solution experiments used 0.03% C_12_E_9_. Other reaction components were at concentrations stated in the text.

Depending on the *K*_d_, data were fitted either to a one-site ligandbinding equation (eqn 1), or to a one-site quadratic tight ligand-binding equation (when the enzyme concentration approaches the binding affinity; eqn 2), defined as:

(1) $$v=\frac{V_{\max}\cdot \left[L\right]}{K_{d}+\left[L\right]}+\mathrm{background}$$

where *ν is* equal to the enzyme velocity, *V*_max_ is the total capacity of the ligand-associated ATPase stimulation, [L] is the ligand concentration and *K*_d_ is the dissociation constant for SecA–ligand. The ‘background’ is the ATPase activity without added ligand.

(2) $$\begin{array}{ccc} v & =\; & V_{\max}\cdot \frac{\left[L\right]\;+\;\left[E_{0}\right]\;+\;K_{d}\;-\;\sqrt{{\left(\left[L\right]\;+\;\left[E_{0}\right]\;+\;K_{d}\right)}^{2}\;-\;4\;\cdot \;\left[E_{0}\right]\;\cdot \;\left[L\right]}}{2\;\cdot \;[E_{0}]}\\ & & +\;\mathrm{background} \end{array}$$

where [*E*_0_] is the total SecA concentration. All data were fitted using GraFit (Erithacus).

Protein translocation assays were performed as described previously [19]. Briefly, SecA, ATP and proOmpA_Δcys_ were incubated with vesicles reconstituted with purified SecYEG. Successful translocation of the substrate into the interior was monitored by protease protection and Western blotting for proOmpA_Δcys_.

### Insertion of the SecA 2HF into SecY_268Fl_EG

SecY_268Fl_EG was made by covalent modification of SecY_K268C_EG with fluorescein [24]. Fluorescence quenching of SecY_268Fl_EG was monitored in a Jobin Yvon Fluorolog (Horiba Scientific) using an excitation wavelength of 495 nm, and an emission wavelength of 515 nm. SecY_268Fl_EG (5 nM) and 1 mM p[NH]ppA were added to 1 ml of fluorescence buffer [20 mM Tris/HCl (pH 8), 130 mM NaCl, 10% glycerol, 2 mM MgCl_2_ and 0.03% C_12_E_9_]. SecA_Δcys_, SecA_D337C/E806C_ or SecA_D337C/K482C_ and their corresponding released counterparts (+10 mM DTT) were titrated in over a 0–1 μM concentration range, and fluorescence readings were taken 200 s after each SecA addition.

Results were presented as the percentage fluorescence quench relative to baseline, and fitted according to a one-site ligand-binding equation with linear phase (eqn 3) using GraFit (Erithacus).

(3) $$F=F_{\max}\cdot [L]/(K_{d}+[L])+m[L]$$

where *F* is the percentage signal change, *F*_max_ is the maximum signal change, [L] is the ligand concentration and *K*_d_ is the dissociation constant for SecA–ligand. *m* represents the gradient of a non-saturable linear increase which was subsequently subtracted from the data.

### Supplementary online data

The Supplementary Online Data (at http://www.BiochemJ.org/bj/449/bj4490695add.htm) describes determination of *K*_m_ and *k*_cat_ for the mutants (Supplementary Figure S1). A further explanation of the model (see Figure 7) shows how the translocation process is affected by the various cross-links (Supplementary Figure S2).

## RESULTS

### The PPXD of SecA moves freely within SecA dimers

In order to understand the connotations of the mobility of the PPXD we immobilized it by various intramolecular disulfide bonds. Site-specific cysteine residues were incorporated into an otherwise cysteine-free background to lock the clamp in the open (SecA_D337C/E806C_) or closed (SecA_D337C/K482C_) positions (Figures 1A and 1B). SDS/PAGE resolved the intramolecular cross-links, which were present at approximately 50% of the total purified protein (Figure 1C, lanes 3 and 6); in both cases the PPXD could be predominantly cross-linked or released, by exposure to either oxidizing or reducing agents. Thus the two different cross-linked forms of SecA, with the clamp held open and shut, could be effectively purified for further analysis (Figure 1C, lanes 4 and 7) and compared with their counterparts released by reduction with DTT (Figure 1C, lanes 5 and 8).

SecA without cysteine residues (SecA_Δcys_) behaves similarly to the wild-type [25] and was used as a control. The SecA_Δcys_ protein had the same gel mobility under either oxidizing or reducing conditions, and was indistinguishable from the reduced forms of the double-cysteine mutants (Figure 1C, lanes 1, 2, 5 and 8).

Size-exclusion chromatography was then used to distinguish large changes in the globular structure or oligomeric state of the cross-linked enzyme. In all cases when the clamp was released by DTT (Figure 1C, lanes 5 and 8), or in the control (Figure 1C, lanes 1 and 2), the proteins eluted at the same position (Figures 1D–1F, broken lines), with an apparent molecular mass of ~250 kDa, characteristic of SecA dimers. When the clamp was fixed in either the open or closed states (Figure 1C, lanes 4 and 7) the decrease in elution volume indicated a large conformational change (Figures 1E and 1F, continuous lines). These effects are incompatible with the formation of SecA monomers, which instead would have resulted in an increase in the elution volume. Therefore the location of the PPXD is apparently not dependent on the oligomeric state of SecA.

### The transposition of the PPXD from the HWD to NBD2 increases the ATPase activity of SecA and its affinity for SecYEG

**Figure 2:**
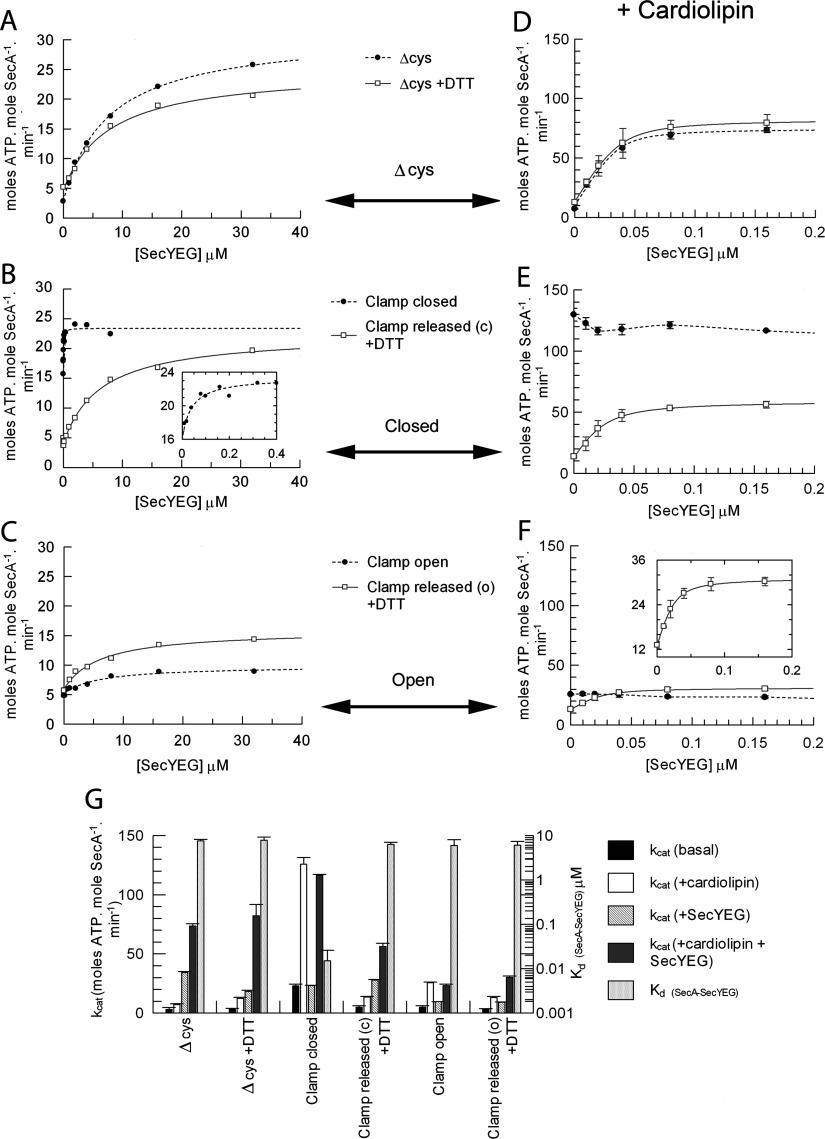
Analysis of SecA ATPase activity and binding affinity for SecYEG Steady-state ATPase activity of 0.15 μM SecA in TKM buffer in the presence of 1 mM ATP and increasing concentrations of purified detergent-solubilized SecYEG. Data were fitted to a ligand-binding equation and the parameters are shown in Supplementary Table S1 (at http://www.BiochemJ.org/bj/449/bj4490695add.htm), columns 1 and 2. (**A**) SecA_Δcys_ in the absence (broken trace) and presence (continuous trace) of 10 mM DTT. (**B**) SecA_D337C/K482C_ cross-linked with the clamp closed (broken trace; no DTT) and with the clamp released from the closed (c) state (continuous trace; 10 mM DTT). (**C**) SecA_D337C/E806C_ cross-linked with the clamp open (broken trace; no DTT) and with the clamp released from the open (o) state (continuous trace; 10 mM DTT). (**D**–**F**) Experiments shown in (**A**–**C**) were repeated in the presence of 40 μM CL. (**G**) The end point *k*_cat_ values under the conditions described, along with the *K*_d_ of SecA binding SecYEG, are shown in the histogram (also in Supplementary Table S1, columns 3 and 4).

The ATPase activity of SecA and its stimulation by SecYEG were tested with the clamp fixed in the open and closed positions. The activation and apparent affinity of the SecA_Δcys_ control for SecYEG was similar to the wild-type [19] and unaffected by DTT (Figures 2A and 2G). However, when the clamp was held in the closed position, the basal ATPase activity (without SecYEG) was increased ~5-fold compared with the SecA_Δcys_ control (0 μM point in Figure 2B, and Figure 2G). In the presence of saturating SecYEG, the stimulation reached the same level as the control; however, the apparent affinity was considerably higher, ~500-fold (Figures 2B and 2G and Supplementary Table S1 at http://www.BiochemJ.org/bj/449/bj4490695add.htm, columns 1 and 2). The effects on both basal activity and affinity for SecYEG were reversed on release of the clamp by DTT. In contrast, the basal activity of the mutant with the clamp locked open was unchanged; its activation by SecYEG was reduced and the binding affinity was unaffected (Figures 2C and 2G and Supplementary Table S1, columns 1 and 2).

The SecYEG-stimulated ATPase activity of SecA (increased *k*_cat_) is also accompanied by a decrease in affinity for ATP (increased *K*_m_) [19,23]. When the PPXD was fixed adjacent to NBD2, with the clamp closed, the kinetic parameters (*K*_m_~1.7 μM and *k*_cat_~9 min^−1^) corresponded to the uncross-linked enzyme bound and activated by SecYEG (*K*_m_~1.8 μM and *k*_cat_~8.5 min^−1^) (Supplementary Figure S1 and Supplementary Table S1 at http://www.BiochemJ.org/bj/449/bj4490695add.htm). Upon release of the cross-link, the kinetic properties returned to the ground state (*K*_m_~0.1 μM and *k*_cat_~0.5 min^−1^) and the dependence on SecYEG for activation was re-established. When the PPXD was fixed adjacent to the HWD, with the clamp open, the kinetics mirrored the inactive enzyme (*K*_m_~0.1 μM and *k*_cat_~0.4 min^−1^) (Supplementary Figure S1 and Supplementary Table S1). Therefore the relocation of the PPXD and contact with NBD2 communicates a change in turnover and affinity for ATP, corresponding to the activation by SecYEG.

### The action of CL (cardiolipin) (diphosphatidylglycerol) with respect to PPXD

CL is a ubiquitous negatively charged phospholipid, required for protein translocation and a range of other essential energy-transducing membrane-transport systems (see [26] and references therein). It stabilizes SecYEG dimers to form a high-affinity binding surface for SecA and confers its ability to activate the ATPase activity. These effects were re-investigated with respect to the different positions occupied by the PPXD within SecA. As expected, exposure of SecA_Δcys_ to CL increased the ATPase activity and tightened the affinity for SecYEG (Figures 2D and 2G and Supplementary Table S1, columns 3 and 4).

SecA with the clamp fixed closed was fully activated in the presence of only CL and the ATPase activity was not further stimulated by the addition of SecYEG (Figures 2E and 2G). When the clamp was fixed open, the basal activity remained low and there was no stimulation by SecYEG (Figures 2F and 2G). Releasing the constraints with DTT restored the wild-type (SecA_Δcys_) behaviour: tight binding of SecYEG causing stimulation of the ATPase activity (Figures 2E–2G and Supplementary Table S1).

The results show that closing the clamp in the presence of CL is sufficient to prime the ATPase for translocation, even in the absence of SecYEG.

### The insertion of the 2HF of SecA is not dependent on the location of the PPXD

**Figure 3:**
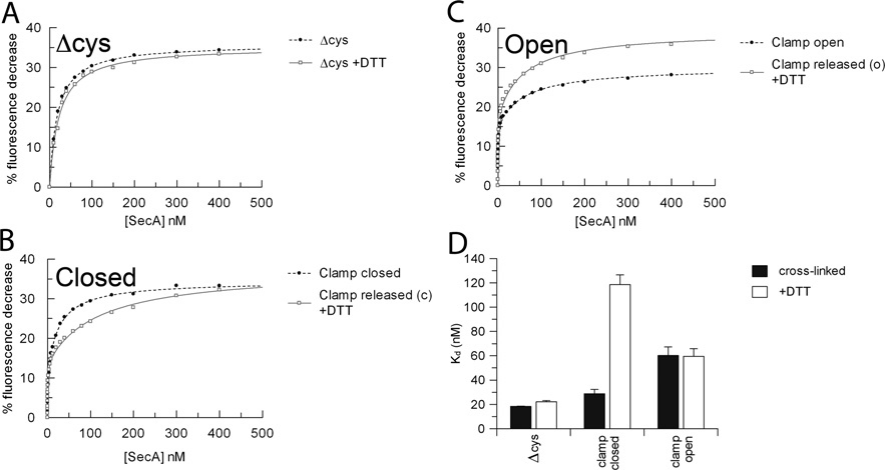
Measurement of the binding of the SecA 2HF to SecY_268Fl_EG SecA was titrated into a solution containing 5 nM SecY_268Fl_EG and the fluorescence quench expressed as the percentage fluorescence decrease. (**A**) SecA_Δcys_ in the absence (broken trace) and presence (continuous trace) of 10 mM DTT. (**B**) SecA_D337C/K482C_ in the absence (broken trace; clamp closed) and presence [continuous trace; clamp released from closed (c)] of 10 mM DTT. (**C**) SecA_D337C/E806C_ in the absence (broken trace; clamp open) and presence [continuous trace; clamp released from open (o)] of DTT. (**D**) Parameters from the fits are shown in the histogram (also in Supplementary Table S1 at http://www.BiochemJ.org/bj/449/bj4490695add.htm, columns 7 and 8).

The insertion of the 2HF into SecYEG was monitored by fluorescence. The ATP-dependent conformational change reported by SecY_268Fl_EG [24] was induced by all versions of SecA, irrespective of the position of the PPXD (Figure 3). Therefore the positioning of the 2HF in the channel is independent of the state of the clamp. The mock oxidized and reduced SecA_Δcys_ controls both bound with the same affinity (Figures 3A and 3D). However, in the active state when the clamp was locked closed, the affinity was ~4-fold tighter compared with the counterpart released by DTT (Figures 3B and 3D). Conversely, when the clamp was locked in the open state the affinity was unchanged with respect to the unleashed enzyme (Figures 3C and 3D).

### Immobilization of the PPXD disables protein translocation

**Figure 4:**
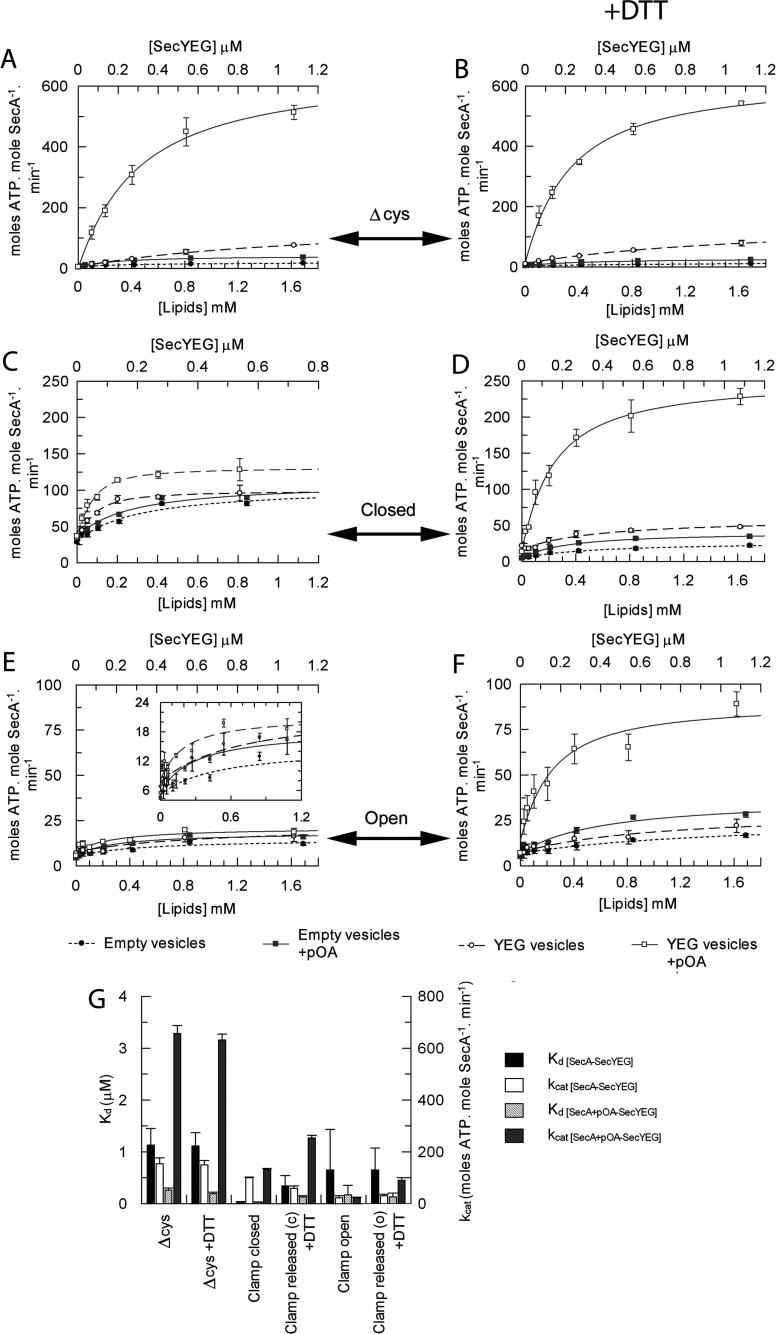
Analysis of the effect of proOmpA on the binding affinity between SecA and SecYEG The steady-state ATPase activity of 80 nM SecA in TKM buffer was measured in the presence of 1 mM ATP and increasing amounts of either empty vesicles (● and 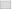, bottom axis) or vesicles reconstituted with purified SecYEG (○ and □, top axis) in the absence or presence of 0.6 μM proOmpA_Δcys_ (circles compared with squares respectively). (**A**) SecA_Δcys_, (**B**) SecA_Δcys_+10 mM DTT, (**C**) SecA_D337C/K482C_ with the clamp locked closed, (**D**) SecA_D337C/K482C_ with the clamp released from closed (c)+DTT, (**E**) SecA_D337C/E806C_ with the clamp locked open, (**F**) SecA_D337C/E806C_ with the clamp released from the open state (o)+DTT. (**G**) Data were fitted to a ligand-binding equation and the parameters are shown in the histogram (also in Supplementary Table S1 at http://www.BiochemJ.org/bj/449/bj4490695add.htm, columns 9–12).

The effect of restraining SecA was assessed with respect to ATP-driven translocation of pre-protein. Vesicles reconstituted with or without SecYEG were titrated into reaction mixtures containing SecA, ATP and, where indicated, the pre-protein substrate proOmpA (Figure 4). The ATPase activity of SecA (or SecA_Δcys_) is stimulated by vesicles harbouring SecYEG much more effectively than the detergent-solubilized complex (Figures 2A, 4A and 4G, and Supplementary Table S1), as expected [19]. In the presence of proOmpA there was a further ~5-fold increase in the affinity of SecA (SecA_Δcys_) for SecYEG and added stimulation of the ATPase activity, associated with translocation into the vesicle interior [19]. This was true of both oxidized and reduced versions of the control SecA_Δcys_ (Figures 4A, 4B and 4G, and Supplementary Table S1, columns 9–12).

SecA with the clamp shut associated with membrane-bound SecYEG more tightly than the corresponding reduced form and the controls (Figures 4A, 4C and 4G), consistent with the increase in affinity also seen in solution (Figure 2B). However, the presence of proOmpA had little further effect on the ATPase activity or the affinity for SecYEG (Figures 4C and 4G, and Supplementary Table S1, columns 9 and 11). Release of the cross-link by DTT restored the expected effect: a large stimulation in the ATPase activity and increased affinity of SecA for SecYEG (Figures 4D and 4G). The version of SecA with its clamp locked open, as expected, did not respond appreciably to vesicles containing SecYEG or to pre-protein (Figure 4E), but was reactivated upon release of the PPXD with DTT (Figure 4F).

**Figure 5:**
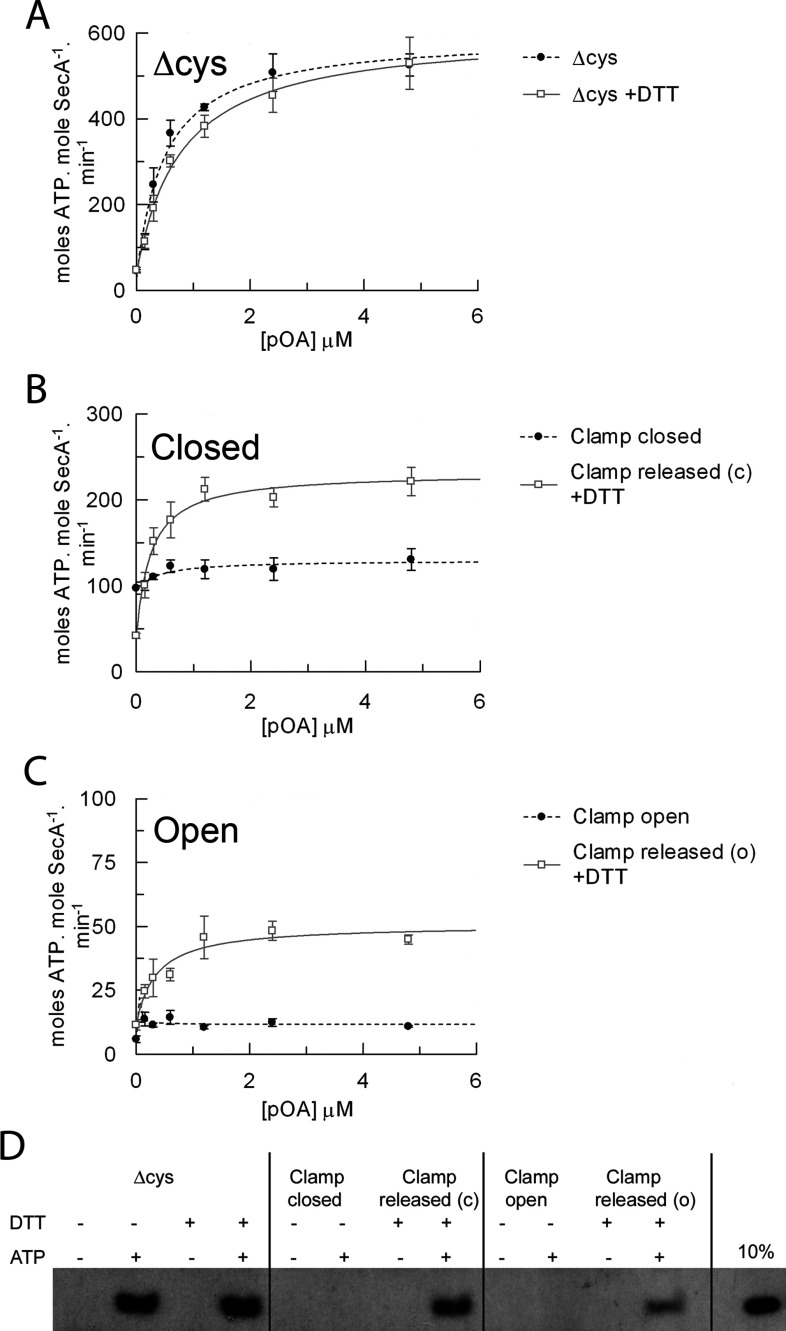
Intramolecular immobilization of the PPXD within SecA inhibits protein translocation Steady-state ATPase activity of 80 nM SecA in TKM buffer in the presence of 1 mM ATP and 1.08 μM SecYEG reconstituted into *E. coli* polar lipids, and increasing amounts of purified proOmpA_Δcys_. (**A**) SecA_Δcys_ in the absence (broken trace) and presence (continuous trace) of 10 mM DTT. (**B**) SecA_D337C/K482C_ in the absence (broken trace; clamp closed) and presence [continuous trace; clamp released from the closed state (c)] of 10 mM DTT. (**C**) SecA_D337C/E806C_ in the absence (broken trace; clamp open) and presence [continuous trace; clamp released from the open state (o)] of DTT. Data were fitted to a ligand-binding equation and the parameters are shown in Supplementary Table S1 at http://www.BiochemJ.org/bj/449/bj4490695add.htm, columns 13 and 14. (**D**) *In vitro* translocation reactions were carried out from end point ATPase assays. Successfully translocated proOmpA was detected via immunoblot analysis.

Next, the translocation-associated ATPase activity of the preformed SecA–SecYEG membrane–bound complex was monitored following the addition of proOmpA_Δcys_ (Figure 5A). Without addition of proOmpA (0 μM point), with the clamp locked closed, the ATPase activity was elevated (Figure 5B), whereas it was low when locked open (Figure 5C, as seen before in Figures 4C and 4E). In both cases the activity did not significantly change following addition of the substrate (Figures 5B and 5C, and Supplementary Table S1, columns 13 and 14). Stimulation of the ATPase activity by substrate was again rescued upon release of the clamp with DTT.

Finally, the different SecA forms were tested for their ability to transport proOmpA across the membrane. Cross-linking the clamp in either the closed or open position abolished transport activity, whereas release of the PPXD with DTT restored it (Figure 5D). Therefore, in spite of the activation achieved by intramolecular cross-linking the PPXD to NBD2, its formation prevents the productive association of SecA with the pre-protein.

### Isolation and analysis of the SecA–SecYEG complex with the PPXD locked in the activated state

**Figure 6:**
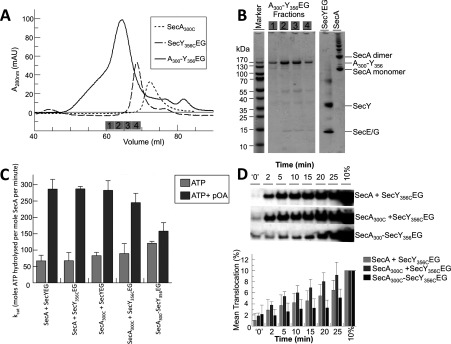
Purification and analysis of the cross-linked SecA–SecYEG complex (**A**) Size-exclusion chromatography: SecY_356C_EG, SecA_300C_ and SecA_300_–Y_356_EG. Fractions analysed by SDS/PAGE in (**B**) are indicated by the numbered grey boxes. (**B**) SDS/PAGE of the purified cross-linked SecA_300_–Y_356_EG complex contained in adjacent fractions eluting from the final size-exclusion purification step. SecYEG and a SecA sample containing monomers and SDS-resistant dimers (and other higher aggregated states) were run as additional gel markers (not as representatives of the input to the cross-linking experiment). (**C**) Steady-state ATPase activity of 0.3 μM wild-type SecA or SecA_300C_ with saturating (1 μM) wild-type SecYEG or SecY_356C_EG proteoliposomes, or the cross-linked complex SecA_300_–Y_356_EG reconstituted into phospholipid vesicles, with and without 0.7 μM proOmpA. The results were averaged from four independent experiments. (**D**) Top panel: relative levels of translocation for wild-type SecA or SecA_300C_ with SecY_356C_EG proteoliposomes, or the cross-linked complex (SecA_300_–Y_356_EG) proteoliposomes over 25 min, analysed by anti-proOmpA immunoblot. *t*=‘0’ represents translocation achieved after initial mixing of reaction components, approximately 10 s. The blot is representative of *n*=5, which were quantified using ImageJ (NIH) software (bottom panel).

Next, we investigated the behaviour of the SecA–SecYEG complex with the PPXD immobilized in the activated position, adjacent to the NBDs [7], by an intermolecular disulfide bridge to the fifth cytosolic loop of SecY (C5; SecA_S300C_ and SecY_I356C_; Figure 1B). Purified SecA_S300C_, p[NH]ppA and crude membranes containing overexpressed SecY_I356C_EG were mixed and oxidized to form the cross-linked complex, which was purified successively by successive Ni^2+^-chelating, ion-exchange and gel-filtration chromatography steps (Figures 6A and 6B). Analysis of the latter by SDS/PAGE indicated that the complex was largely free of the uncross-linked components, which elute later from the column (shown for comparison in Figure 6A).

The complex was then reconstituted into phospholipid vesicles and its ATPase ability and protein transport activity were evaluated. In respect to ATP turnover and stimulation by SecYEG and proOmpA_Δcys_, the individual single mutants (uncross-linked) behaved similarly to the wild-type (Figure 6C). When cross-linked, the uncoupled ATPase (in the absence of pre-protein) was elevated to similar levels observed when the PPXD was cross-linked to NBD2 (Figure 2C and 6C). However, in marked contrast with the previous experiment, the addition of proOmpA_Δcys_ resulted in a small, but significant, stimulation of ATPase activity and successful translocation (Figure 6D). The polypeptide transport activity was reduced by approximately one-half compared with the uncross-linked material, possibly due to partial restrictions imposed by the cross-link. The results demonstrate that, following its transit from the HWD to the NBD2, the PPXD does not move back to its resting state until translocation is complete.

## DISCUSSION

The consequences of the location and restriction of the PPXD, and in a related study the 2HF [22], of SecA have been used to explore the dynamic mechanism of ATP-driven protein transport. This has been achieved by the incorporation of unique cysteine pairs for the formation of intra- and inter-molecular disulfide bonds by oxidation. The mutants used in the present study have afforded minor changes in the affinity of SecA for SecYEG (Figure 3) as well as the ATPase activity (Figures 4 and 5). This is perhaps not very surprising given that the mutations themselves are at the interface between the PPXD and the NBDs of SecA and cytosolic surface of SecY. The key findings of the present study were related by much more profound consequences on the affinity and activation of the enzyme upon the formation of each disulfide bond. Significantly, these changes are reversed upon the release of the restrictions by a reducing agent.

The PPXD alternates between the HWD and NBDs within the intact SecA dimer. These transitions do not appear to be coupled to ATP hydrolysis, in agreement with the nucleotide-independent conformational change of this domain seen in the SecA monomer [17].

The formation of SecA monomers following the initiation of translocation (see the Introduction) exposes the SecYEG-binding site, including the 2HF of the HSD [7] (Figure 1B). This interaction has been monitored by an extrinsic fluorescent probe in SecY [24], which was elicited irrespectively of the position of the PPXD. Therefore the dissociation of SecA dimers is not strictly dependent on the position of the PPXD.

The relocation of the PPXD away from the HWD results in a switch in the kinetic properties, akin to those observed in the presence of SecYEG, together with an increase in affinity for the protein channel. Evidently then, the initial contact of SecA with SecYEG promotes the transposition of the PPXD, most probably due to its interaction with cytoplasmic loop C5 of SecY, between TMSs 8 and 9 [7]. In this position the PPXD is in direct contact with the NBD to activate the enzyme, presumably by promoting the release of ADP, which is the rate-limiting step of the ATP hydrolytic cycle [19]. The low ATPase activity observed when the PPXD is adjacent to the HWD, with the clamp open, reflects the stability of the complex of the enzyme with ADP, in agreement with the known tight association between the PPXD and HWD in this state [16,17].

The cross-link between the PPXD and NBD2 activates the ATPase, but inhibits transport. Therefore the cross-link may have blocked the intercalation path for the pre-protein into the translocation clamp (see also below). Changing the attachment to the interaction site with SecY-C5 retained the transport activity. Therefore the PPXD does not need to move significantly once translocation is underway. The large movement of the latter is presumably only required during substrate binding and the initiation step (see below).

**Figure 7:**
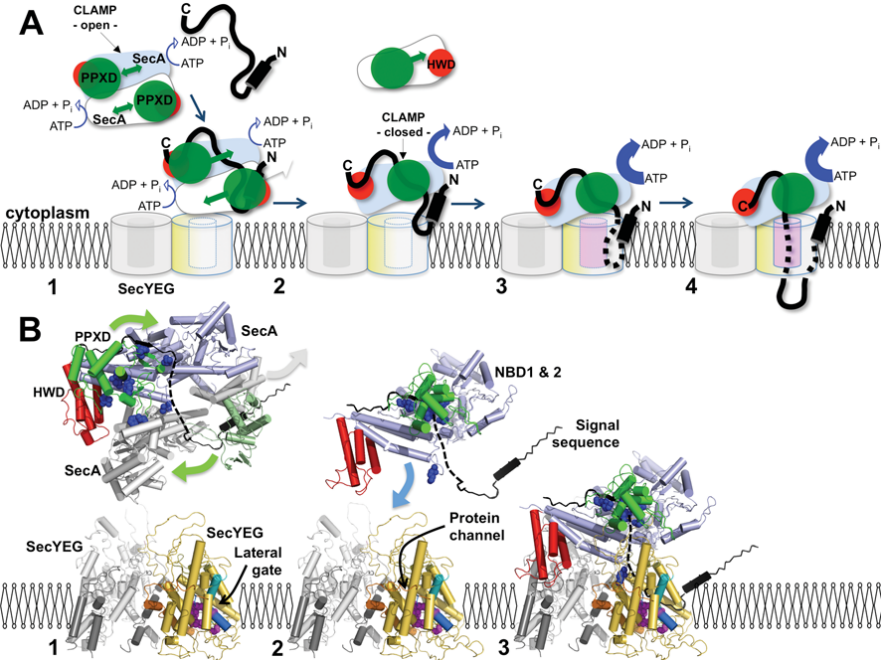
Model depicting the role of the PPXD in protein translocation (**A**) Schematic diagram and (**B**) structural (cartoon) representation of various states of the translocation cycle of SecA and SecYEG. SecYEG: non-translocation complex (grey), translocating complex (yellow), within (**B**) TMS2b (cyan cylinder) and TMS7 (blue cylinder) of the lateral gate, as well as the residues that contact substrate (magenta space-fill) [20]. SecA: two monomers of the dimer (pale blue and white), PPXD (green), HWD (red) and in (**B**) residues that contact substrate (blue space-fill) [15]. Pre-protein (black) with the N-terminal signal sequence (black cylinder). Stage (1) initiation: SecA dimers (low ATPase activity [23]) engage SecYEG. The mobility of the PPXD permits the binding of the pre-protein between the PPXD and the HWD. In (**B**) the structure of the SecA dimer is shown [6], where the lower monomer has been replaced by SecA (white with PPXD in pale green) bound to the signal sequence (black) [14]. In the upper monomer the C-terminus is shown in black, which may occupy the pre-protein-binding site [27]. The broken black line connects this C-terminal stretch to the signal sequence, describing a possible continuous binding groove for the pre-protein. SecYEG here and in subsequent stages (2) and (3) was modelled on the structure of the membrane-bound complex [4,9]. Stage (2) activation: dissociation of SecA [18] exposes the SecYEG-binding site of SecA [7]. The relocation of the PPXD serves to increase the affinity for SecYEG, activate the ATPase activity [19], release the signal sequence from the departing SecA and close the clamp about the pre-protein. In (**B**) the dissociated SecA is removed but the signal sequence is retained. The remaining monomeric enzyme has been replaced by the activated version seen in the SecA–SecYEG complex [7], with the PPXD adjacent to the NBDs to trap the pre-protein. Stage (3) insertion: the resultant association of the monomeric SecA with SecYEG displays the signal sequence to the binding site at the lateral gate of SecY [8,28]. The binding of which unlocks SecYEG [8] to promote the intercalation of the translocation substrate into the protein channel and the full activation of the ATPase [19]. In (**B**) the model of the membrane-bound translocon is shown [7,9] retaining the pre-protein from the previous alignment. Stage (4) transport: the trapped and inserted pre-protein passes through the membrane via a single SecYEG complex.

On the basis of these results and a re-evaluation of the structures of SecA and SecYEG, including those associated with pre-protein peptide mimics [8,14], a model for the initiation of translocation has been formulated (Figure 7). The pre-protein-binding sites on SecA have been identified by cross-linking [13,15], NMR [14] and X-ray crystallography [27] and allow us to partially map the pre-protein-binding site on SecA (Figure 7: stage 1, black polypeptide). The signal sequence binds between the PPXD and HWD [14], and a site for the mature protein has been localized by the C-terminus of SecA [6], proposed to mimic the substrate by β-augmentation of the two strands connecting the PPXD to NBD1 [27]. The relocation of the PPXD would effectively release the signal sequence, encapsulate the mature regions of the pre-protein and increase the affinity for the SecY complex (Figure 7: stage 1–2). The subsequent SecYEG-induced dissociation of SecA dimers [18,24] would expose the pre-protein to SecY (Figure 7: stage 2). The association of the well-placed signal sequence would then unlock the SecY complex and facilitate the intercalation of the mature region of the pre-protein [8] (Figure 7: stage 2–3). This mechanism avoids the need to transfer the signal sequence from between the PPXD and HWD 60 Å to the other side of the SecA–SecYEG complex. Note also that the model is not absolutely dependent on two copies of SecYEG.

The model explains the effects observed when the PPXD is immobilized by intramolecular cross-links in the resting (clamp open) or activated (clamp closed) states, including why the latter is incapable of forming a productive interaction with pre-protein (Supplementary Figures S2A and S2B at http://www.BiochemJ.org/bj/449/bj4490695add.htm). In contrast, the alternatively activated enzyme, with the PPXD cross-linked to SecY, is fully primed and capable of pre-protein binding and transport (Supplementary Figure S2C).

The mechanism of ATP-driven energy-transducing systems often relies on large domain movements coupled to changes in the affinity for ATP, ADP and substrate, in this case pre-protein. SecA seems to have adopted a method whereby a large domain movement is involved during the initiation phase for both the activation of the NBDs and the trapping and release of pre-protein.

## Online data

Supplementary data
