# Supplementary material for: The dynamic action of SecA during the initiation of protein translocation
Source: Biochem J. 2013 Jan 9;449(Pt 3):695–705. doi: 10.1042/BJ20121314 (PMC3685266; doi:10.1042/BJ20121314)
Supplement: Supplementary data [file bj4490695add.pdf]

BJ Structure

Biochem. J. (2013) **449**, 695–705 (Printed in Great Britain) doi:10.1042/BJ20121314

online data

SUPPLEMENTARY ONLINE DATA

The dynamic action of SecA during the initiation of protein translocation

Vicki A. M. GOLD[1], Sarah WHITEHOUSE, Alice ROBSON and Ian COLLINSON[2]

School of Biochemistry, University of Bristol, University Walk, Bristol BS8 1TD, U.K.

MATERIALS AND METHODS

Steady-state ATPase and determination of the K_m

Steady-state SecA ATPase measurements were performed as described previously [1,2] with various concentrations of ATP. The K_m and V_{max} (k_{cat}) were determined by fitting the data to the Michaelis–Menten equation (eqn S1)

$$v = \frac{V_{max} \cdot [S]}{K_m + [S]}$$

where v *is* equal to the enzyme velocity, V_{max} is the total capacity of the substrate-associated ATPase stimulation, [S] is the substrate concentration and K_m is the Michaelis constant.

[1] Present address: Max-Planck-Institute of Biophysics, Department of Structural Biology, Max-von-Laue-Strasse 3, D-60438 Frankfurt am Main, Germany

[2] To whom correspondence should be addressed (email ian.collinson@bristol.ac.uk).

© 2013 The Author(s)

The author(s) has paid for this article to be freely available under the terms of the Creative Commons Attribution Licence (CC-BY) (http://creativecommons.org/licenses/by/3.0/) which permits unrestricted use, distribution and reproduction in any medium, provided the original work is properly cited.

Biochemical Journal www.biochemj.org

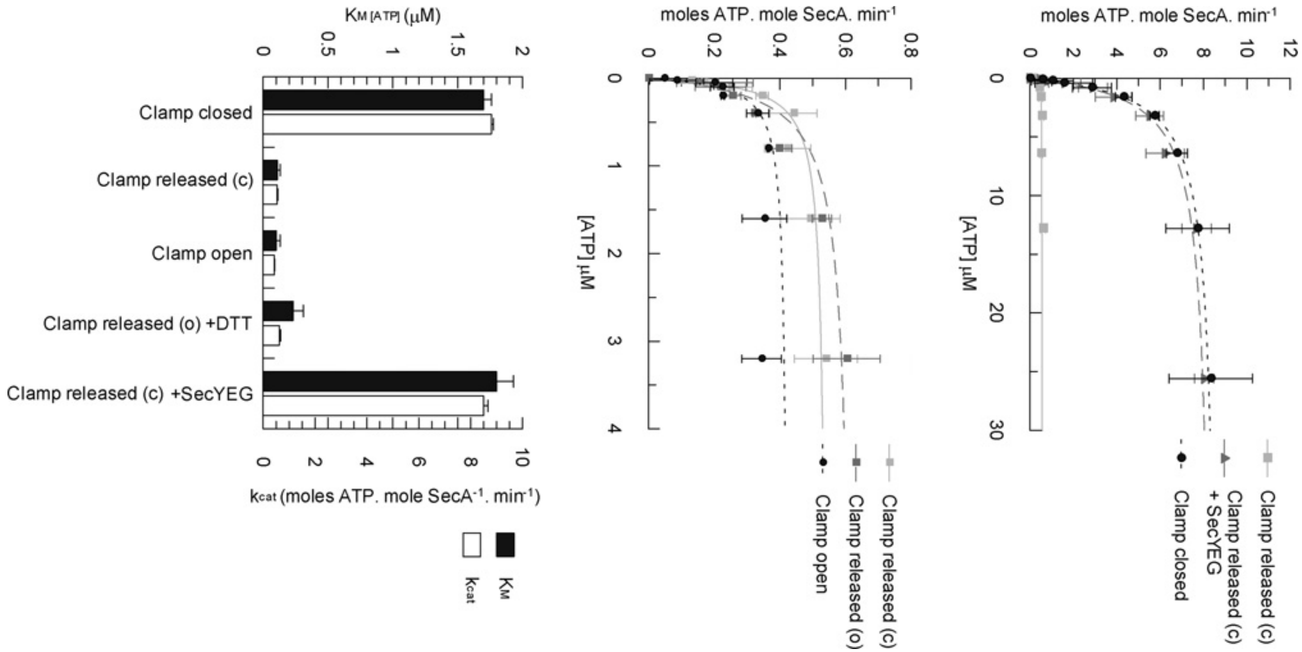

Figure S1 Michaelis–Menten parameters for ATP binding

(A) ATPase rates of SecA (0.75 μM) over a range of ATP concentrations with: the clamp held in the closed state (black dotted trace), the clamp released from the closed (c) state (silver continuous trace, +10 mM DTT) and the same released form in the presence of saturating SecYEG (detergent solubilized; 16 μM) (grey dashed trace). **(B)** ATPase assays were performed as in **(A)** using SecA with: the clamp open (black dotted trace) and released from the either the closed (c) or open (o) states (silver continuous trace and grey dashed trace respectively). **(C)** Data in **(A)** and **(B)** were fitted to the Michaelis–Menten equation and the parameters are shown in the histogram (also in Table S1, columns 5 and 6).

Table S1 Kinetic parameters

Various parameters derived from data fits (see individual Figure legends in the main text). The data for the clamp-closed SecA are shown in bold to emphasize the significant differences in this version. Errors represent S.D. values from the fit.

	SecA–SecYEG$_{soluble}$		SecA–SecYEG$_{CL}$		SecA–ATP		SecA$_{(2HF)}$–SecY$_{K268F}$EG$_{soluble}$		SecA–SecYEG$_{vesicles}$		[SecA-pOA]–SecYEG$_{vesicles}$		[SecA-SecYEG$_{vesicles}$]–pOA	
Condition	1 K_d (μM)	2 k_{cat}	3 K_d (nM)	4 k_{cat}	5 K_m (μM)	6 k_{cat}	7 K_d (nM)	8 ΔFI (%)	9 K_d (μM)	10 k_{cat}	11 K_d (μM)	12 k_{cat}	13 K_d (μM)	14 k_{cat}
Δcys	7.6 ± 0.6	31.4 ± 0.6	3.7 ± 1.9	74.9 ± 3.5	–	–	18.4 ± 0.6	35.3 ± 0.2	1.13 ± 0.32	154.2 ± 22.0	0.26 ± 0.04	656.4 ± 31.0	0.50 ± 0.12	605.8 ± 38.3
Δcys + DTT	7.8 ± 1.4	25.0 ± 1.1	5.6 ± 0.6	82.7 ± 0.9	–	–	20.6 ± 2.1	36.0 ± 1.3	1.11 ± 0.26	149.4 ± 17.2	0.19 ± 0.03	631.6 ± 22.6	0.78 ± 0.10	604.6 ± 20.2
**Clamp closed**	**0.015 ± 0.011**	**23.2 ± 0.6**	–	–	**1.7 ± 0.06**	**8.8 ± 0.08**	**28.6 ± 5.0**	**20.9 ± 1.4**	**0.029 ± 0.007**	**99.7 ± 3.0**	**0.025 ± 0.007**	**131.7 ± 4.5**	–	–
Clamp released (c)	6.3 ± 0.7	22.5 ± 0.7	8.8 ± 2.1	59.1 ± 1.9	0.11 ± 0.02	0.54 ± 0.02	117.7 ± 12.7	23.5 ± 0.8	0.34 ± 0.20	59.4 ± 8.2	0.13 ± 0.02	253.6 ± 10.0	0.24 ± 0.06	231.6 ± 13.3
Clamp open	5.9 ± 2.1	9.9 ± 0.5	–	–	0.10 ± 0.03	0.43 ± 0.02	60.9 ± 10.0	15.0 ± 0.6	0.65 ± 0.78	22.5 ± 7.8	0.17 ± 0.18	21.1 ± 3.5	–	–
Clamp released (o)	6.0 ± 1.4	15.9 ± 0.6	6.5 ± 0.9	31.2 ± 0.4	0.23 ± 0.08	0.63 ± 0.05	59.5 ± 6.4	21.3 ± 0.6	0.65 ± 0.42	29.9 ± 6.4	0.13 ± 0.07	90.3 ± 9.7	0.35 ± 0.15	50.5 ± 4.7
Clamp released (c) + SecYEG	–	–	–	–	1.8 ± 0.13	8.5 ± 0.17	–	–	–	–	–	–	–	–

© The Authors Journal compilation © 2013 Biochemical Society

© 2013 The Author(s)
The author(s) has paid for this article to be freely available under the terms of the Creative Commons Attribution Licence (CC-BY) (http://creativecommons.org/licenses/by/3.0/) which permits unrestricted use, distribution and reproduction in any medium, provided the original work is properly cited.

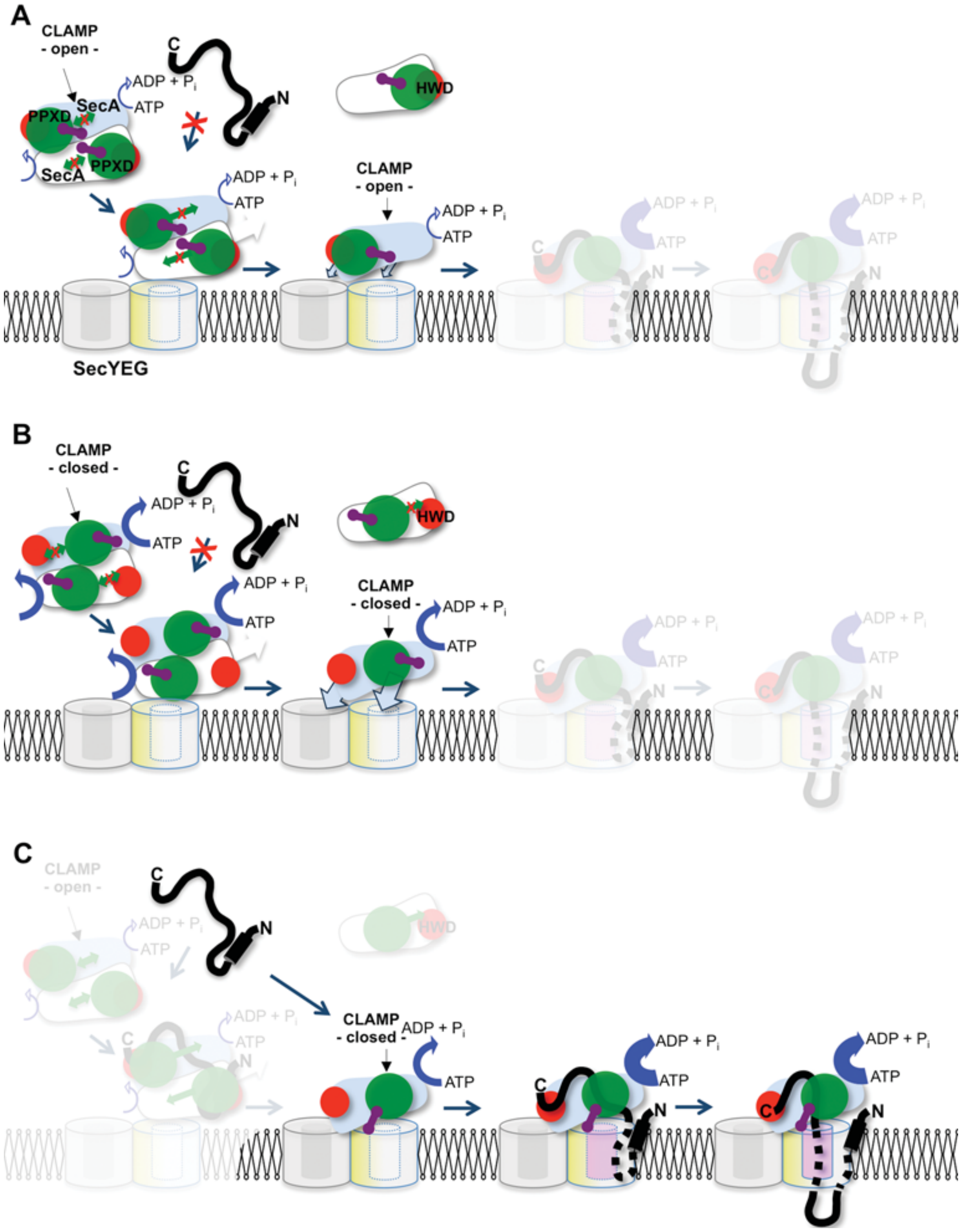

Figure S2 The consequences of the immobilization of the PPXD within SecA to protein transport

The model of pre-protein transport (Figure 7A of the main text) has been modified to incorporate the intra- and inter-molecular disulfide bonds, and to explain their effect on the activity. Colour co-ordination and labelling is as in Figure 7 of the main text. Stages of the mechanism precluded as a result have been fogged out. (**A**) When the PPXD is cross-linked (purple bar) to the HSD the clamp is permanently held open. This prevents the activation of the ATPase activity (thin blue arrows), maintains a low affinity for SecYEG (small pale blue arrows) and prevents association with the pre-protein. (**B**) Fixing the PPXD to NBD2, holding the clamp closed, activates the ATPase activity (blue arrows), brings about a high-affinity association with SecYEG (large pale blue arrows), but prevents the engagement of pre-protein. (**C**) The pre-activated cross-linked SecA–SecYEG complex is primed for translocation and capable of pre-protein intercalation and ATP-driven (thick blue arrows) translocation.

REFERENCES

1 Robson, A., Gold, V. A., Hodson, S., Clarke, A. R. and Collinson, I. (2009) Energy transduction in protein transport and the ATP hydrolytic cycle of SecA. Proc. Natl. Acad. Sci. U.S.A. **106**, 5111–5116

2 Deville, K., Gold, V. A., Robson, A., Whitehouse, S., Sessions, R. B., Baldwin, S. A., Radford, S. E. and Collinson, I. (2011) The oligomeric state and arrangement of the active bacterial translocon. J. Biol. Chem. **286**, 4659–4669

Received 20 August 2012/15 October 2012; accepted 5 November 2012
Published as BJ Immediate Publication 5 November 2012, doi:10.1042/BJ20121314

© 2013 The Author(s)
The author(s) has paid for this article to be freely available under the terms of the Creative Commons Attribution Licence (CC-BY) (http://creativecommons.org/licenses/by/3.0/) which permits unrestricted use, distribution and reproduction in any medium, provided the original work is properly cited.
